# Supplementary material for: MetaQTL: a package of new computational methods for the meta-analysis of QTL mapping experiments
Source: BMC Bioinformatics. 2007 Feb 8;8:49. doi: 10.1186/1471-2105-8-49 (PMC1808479; doi:10.1186/1471-2105-8-49)
Supplement: Additional File 7 — MetaQTL Package : jar file and tutorial. This Zip archive contains both the MetaQTL JAR file and the files of the tutorial. [file 1471-2105-8-49-S7.zip › org.inra.metaqtl/doc/org/metaqtl/graph/MetaGraphPar.html]

MetaGraphPar


|  |  |  |
| --- | --- | --- |
| **Overview** \| **Package** \| **Class** \| **Use** \| **Tree** \| **Deprecated** \| **Index** \| **Help** | |  |
| **PREV CLASS**   **NEXT CLASS** | **FRAMES**    **NO FRAMES**     **All Classes** |  |
| SUMMARY: NESTED \| FIELD \| CONSTR \| METHOD | DETAIL: FIELD \| CONSTR \| METHOD |  |


---


## org.metaqtl.graph Class MetaGraphPar

```
java.lang.Object
  org.metaqtl.graph.MetaGraphPar
```

---

``` public final class MetaGraphPar extends java.lang.Object ```

---

| **Field Summary** | |
| --- | --- |
| `static java.awt.Color` | `BACKGROUND_COLOR`             The color of the backgroud. |
| `static int` | `CHROM_ALIGN_MODE` |
| `static double` | `CHROM_DISTANCE_SCALE` |
| `static double` | `CHROM_FLANKING_CEX`             The default cex parameter for flanking regions at the top and the end of the chromosome. |
| `static java.awt.Font` | `CHROM_NAME_FONT` |
| `static double` | `CHROM_NAME_HSPACE` |
| `static java.awt.Stroke` | `CHROM_STROKE`             The stroke of the chromosome shape. |
| `static java.awt.Color` | `CHROM_STROKE_COLOR`             The color of the stroke of the Chromosome shape. |
| `static java.awt.Color` | `CHROM_TICK_COLOR`             The color of the marker ticks on the chromosome. |
| `static java.awt.Stroke` | `CHROM_TICK_STROKE` |
| `static double` | `CHROM_TICK_WIDTH_1`             The width of the marker ticks. |
| `static double` | `CHROM_TICK_WIDTH_2` |
| `static double` | `CHROM_TICK_WIDTH_3` |
| `static double` | `CHROM_WIDTH`             The width of the chromosome. |
| `static double` | `COMMON_STROKE_WIDTH` |
| `static double` | `DEFAULT_HEIGHT`             The default height. |
| `static double` | `DEFAULT_WIDTH`             The default width. |
| `static double` | `LAYER_HSPACE`             The horizontal space between layers. |
| `static double` | `LAYER_VSPACE`             The vertical space between layers. |
| `static double` | `LEGEND_BOX_CEX` |
| `static java.awt.Font` | `LEGEND_FONT` |
| `static double` | `LEGEND_GRAD_HEIGHT` |
| `static double` | `LEGEND_GRAD_WIDTH` |
| `static double` | `LEGEND_HSPACE` |
| `static double` | `LEGEND_PART_HEIGHT` |
| `static double` | `LEGEND_PART_WIDTH` |
| `static int` | `LEGEND_SCALE_UNIT` |
| `static double` | `MAKER_NAME_VSPACE` |
| `static java.awt.Font` | `MARKER_NAME_FONT` |
| `static java.awt.Font` | `MARKER_POSITION_FONT` |
| `static java.awt.Color` | `NEG_COMMON_COLOR` |
| `static java.awt.Color` | `POS_COMMON_COLOR` |
| `static int` | `PROBA_BIN` |
| `static java.awt.Color` | `PROBA_FROM_COLOR` |
| `static java.awt.Color[]` | `PROBA_PALETTE` |
| `static java.awt.Color` | `PROBA_TO_COLOR` |
| `static double` | `QTL_CI_WIDTH` |
| `static double` | `QTL_CI_WIDTH_CEX` |
| `static double` | `QTL_HSPACE`             The horizontal space between Qtl shapes. |
| `static java.awt.Color` | `QTL_NAME_COLOR`             The color of the QTL names. |
| `static java.awt.Font` | `QTL_NAME_FONT`             The font used for the Qtl names. |
| `static double` | `QTL_NAME_SPACE` |
| `static double` | `QTL_NAME_VSPACE_CEX`             The space magnification between the Qtl name and the Qtl confidence interval. |
| `static java.awt.Color[]` | `QTL_PALETTE`             The color palete for the QTL partition. |
| `static double` | `QTL_PCH_SIZE` |
| `static double` | `QTL_POS_HEIGHT_CEX` |
| `static double` | `QTL_POS_WIDTH_CEX` |
| `static double` | `QTL_PROB_WIDTH` |
| `static double` | `QTL_TICK_WIDTH_CEX`             The magnification for the tick of the QTL. |
| `static double` | `QTL_TREE_SCALE` |
| `static double` | `QTL_TREE_SPACE` |
| `static double` | `QTL_VSPACE`             The vertical space between Qtl shapes. |
| `static java.awt.Color` | `SINGLE_COMMON_COLOR` |
| `static double` | `TREE_NODE_WIDTH` |
| `static boolean` | `WITH_CHROM_NAME` |
| `static boolean` | `WITH_COMMON_MARKER` |
| `static boolean` | `WITH_LEGEND` |
| `static boolean` | `WITH_MAP_NAME` |
| `static boolean` | `WITH_MARKER_NAME` |
| `static boolean` | `WITH_MARKER_POSITION` |
| `static boolean` | `WITH_QTL_NAME`             Add the Qtl name. |


| **Method Summary** | |
| --- | --- |
| `static java.lang.String` | `color2string(java.awt.Color color)` |
| `static java.lang.String` | `font2string(java.awt.Font font)` |
| `static java.awt.Color` | `getProbaColor(double d)` |
| `static java.awt.Color` | `getProbaColor(double d, double thresh)` |
| `static void` | `initPalettes()` |
| `static void` | `initProbaPalette()` |
| `static void` | `initQTLPalette()` |
| `static void` | `initQTLPalette(int n)` |
| `static void` | `set(java.lang.String parameter, java.lang.String value)` |
| `static java.awt.Color` | `string2color(java.lang.String value)` |
| `static java.awt.Font` | `string2font(java.lang.String value)` |

**Methods inherited from class java.lang.Object**

`clone, equals, finalize, getClass, hashCode, notify, notifyAll, toString, wait, wait, wait`

**Field Detail**

### LAYER\_HSPACE

```
public static double LAYER_HSPACE
```

:   The horizontal space between layers.

---


### LAYER\_VSPACE

```
public static double LAYER_VSPACE
```

:   The vertical space between layers.

---


### DEFAULT\_WIDTH

```
public static double DEFAULT_WIDTH
```

:   The default width.

---


### DEFAULT\_HEIGHT

```
public static double DEFAULT_HEIGHT
```

:   The default height.

---


### BACKGROUND\_COLOR

```
public static java.awt.Color BACKGROUND_COLOR
```

:   The color of the backgroud. Default is white.

---


### QTL\_VSPACE

```
public static double QTL_VSPACE
```

:   The vertical space between Qtl shapes. Default is 5.0

---


### QTL\_HSPACE

```
public static double QTL_HSPACE
```

:   The horizontal space between Qtl shapes. Default is 5.0

---


### QTL\_TREE\_SPACE

```
public static double QTL_TREE_SPACE
```

---


### TREE\_NODE\_WIDTH

```
public static double TREE_NODE_WIDTH
```

---


### WITH\_QTL\_NAME

```
public static boolean WITH_QTL_NAME
```

:   Add the Qtl name. Default is true

---


### QTL\_NAME\_FONT

```
public static java.awt.Font QTL_NAME_FONT
```

:   The font used for the Qtl names.
    Default is Times with type plain and size 8.

---


### MARKER\_NAME\_FONT

```
public static java.awt.Font MARKER_NAME_FONT
```

---


### MARKER\_POSITION\_FONT

```
public static java.awt.Font MARKER_POSITION_FONT
```

---


### CHROM\_NAME\_FONT

```
public static java.awt.Font CHROM_NAME_FONT
```

---


### QTL\_NAME\_COLOR

```
public static java.awt.Color QTL_NAME_COLOR
```

:   The color of the QTL names. Default is black

---


### QTL\_NAME\_VSPACE\_CEX

```
public static double QTL_NAME_VSPACE_CEX
```

:   The space magnification between the Qtl name and the Qtl confidence
    interval.

---


### WITH\_MAP\_NAME

```
public static boolean WITH_MAP_NAME
```

---


### WITH\_CHROM\_NAME

```
public static boolean WITH_CHROM_NAME
```

---


### QTL\_PCH\_SIZE

```
public static double QTL_PCH_SIZE
```

---


### WITH\_LEGEND

```
public static boolean WITH_LEGEND
```

---


### QTL\_TICK\_WIDTH\_CEX

```
public static double QTL_TICK_WIDTH_CEX
```

:   The magnification for the tick of the QTL.

---


### QTL\_PALETTE

```
public static java.awt.Color[] QTL_PALETTE
```

:   The color palete for the QTL partition.
    Default is null.

---


### PROBA\_PALETTE

```
public static java.awt.Color[] PROBA_PALETTE
```

---


### PROBA\_BIN

```
public static int PROBA_BIN
```

---


### CHROM\_WIDTH

```
public static double CHROM_WIDTH
```

:   The width of the chromosome. Default is 20.0

---


### QTL\_CI\_WIDTH

```
public static double QTL_CI_WIDTH
```

---


### CHROM\_NAME\_HSPACE

```
public static double CHROM_NAME_HSPACE
```

---


### WITH\_MARKER\_NAME

```
public static boolean WITH_MARKER_NAME
```

---


### CHROM\_ALIGN\_MODE

```
public static int CHROM_ALIGN_MODE
```

---


### WITH\_MARKER\_POSITION

```
public static boolean WITH_MARKER_POSITION
```

---


### CHROM\_FLANKING\_CEX

```
public static double CHROM_FLANKING_CEX
```

:   The default cex parameter for flanking regions at the
    top and the end of the chromosome.

---


### CHROM\_STROKE

```
public static java.awt.Stroke CHROM_STROKE
```

:   The stroke of the chromosome shape.
    Default is `BasicStroke`

---


### CHROM\_TICK\_STROKE

```
public static java.awt.Stroke CHROM_TICK_STROKE
```

---


### MAKER\_NAME\_VSPACE

```
public static double MAKER_NAME_VSPACE
```

---


### CHROM\_STROKE\_COLOR

```
public static java.awt.Color CHROM_STROKE_COLOR
```

:   The color of the stroke of the Chromosome shape.
    Default is black.

---


### CHROM\_TICK\_WIDTH\_1

```
public static double CHROM_TICK_WIDTH_1
```

:   The width of the marker ticks.

---


### CHROM\_TICK\_WIDTH\_2

```
public static double CHROM_TICK_WIDTH_2
```

---


### CHROM\_TICK\_WIDTH\_3

```
public static double CHROM_TICK_WIDTH_3
```

---


### QTL\_POS\_HEIGHT\_CEX

```
public static double QTL_POS_HEIGHT_CEX
```

---


### QTL\_POS\_WIDTH\_CEX

```
public static double QTL_POS_WIDTH_CEX
```

---


### CHROM\_TICK\_COLOR

```
public static java.awt.Color CHROM_TICK_COLOR
```

:   The color of the marker ticks on the chromosome.
    Default is black.

---


### QTL\_NAME\_SPACE

```
public static double QTL_NAME_SPACE
```

---


### CHROM\_DISTANCE\_SCALE

```
public static double CHROM_DISTANCE_SCALE
```

---


### QTL\_CI\_WIDTH\_CEX

```
public static double QTL_CI_WIDTH_CEX
```

---


### QTL\_PROB\_WIDTH

```
public static double QTL_PROB_WIDTH
```

---


### LEGEND\_FONT

```
public static java.awt.Font LEGEND_FONT
```

---


### LEGEND\_PART\_HEIGHT

```
public static double LEGEND_PART_HEIGHT
```

---


### LEGEND\_PART\_WIDTH

```
public static double LEGEND_PART_WIDTH
```

---


### LEGEND\_HSPACE

```
public static double LEGEND_HSPACE
```

---


### LEGEND\_BOX\_CEX

```
public static double LEGEND_BOX_CEX
```

---


### LEGEND\_SCALE\_UNIT

```
public static int LEGEND_SCALE_UNIT
```

---


### WITH\_COMMON\_MARKER

```
public static boolean WITH_COMMON_MARKER
```

---


### PROBA\_FROM\_COLOR

```
public static java.awt.Color PROBA_FROM_COLOR
```

---


### PROBA\_TO\_COLOR

```
public static java.awt.Color PROBA_TO_COLOR
```

---


### SINGLE\_COMMON\_COLOR

```
public static java.awt.Color SINGLE_COMMON_COLOR
```

---


### POS\_COMMON\_COLOR

```
public static java.awt.Color POS_COMMON_COLOR
```

---


### NEG\_COMMON\_COLOR

```
public static java.awt.Color NEG_COMMON_COLOR
```

---


### COMMON\_STROKE\_WIDTH

```
public static double COMMON_STROKE_WIDTH
```

---


### LEGEND\_GRAD\_HEIGHT

```
public static double LEGEND_GRAD_HEIGHT
```

---


### LEGEND\_GRAD\_WIDTH

```
public static double LEGEND_GRAD_WIDTH
```

---


### QTL\_TREE\_SCALE

```
public static double QTL_TREE_SCALE
```


**Method Detail**

### set

```
public static void set(java.lang.String parameter,
                       java.lang.String value)
                throws java.lang.IllegalArgumentException
```

:   **Parameters:**: `parameter` -: `value` - **Throws:**: `java.lang.IllegalArgumentException`

---


### string2color

```
public static java.awt.Color string2color(java.lang.String value)
```

:   **Parameters:**: `value` - **Returns:**

---


### color2string

```
public static java.lang.String color2string(java.awt.Color color)
```

---


### string2font

```
public static java.awt.Font string2font(java.lang.String value)
```

:   **Parameters:**: `value` - **Returns:**

---


### font2string

```
public static java.lang.String font2string(java.awt.Font font)
```

:   **Parameters:**: `font` - **Returns:**

---


### getProbaColor

```
public static java.awt.Color getProbaColor(double d)
```

:   **Parameters:**: `d` - **Returns:**

---


### getProbaColor

```
public static java.awt.Color getProbaColor(double d,
                                           double thresh)
```

:   **Parameters:**: `d` - **Returns:**

---


### initProbaPalette

```
public static void initProbaPalette()
```

---


### initQTLPalette

```
public static void initQTLPalette()
```

---


### initQTLPalette

```
public static void initQTLPalette(int n)
```

---


### initPalettes

```
public static void initPalettes()
```


---


|  |  |  |
| --- | --- | --- |
| **Overview** \| **Package** \| **Class** \| **Use** \| **Tree** \| **Deprecated** \| **Index** \| **Help** | |  |
| **PREV CLASS**   **NEXT CLASS** | **FRAMES**    **NO FRAMES**     **All Classes** |  |
| SUMMARY: NESTED \| FIELD \| CONSTR \| METHOD | DETAIL: FIELD \| CONSTR \| METHOD |  |


---
